# Evaluation of the Implementation Process of a Family Systems Nursing Approach in Home Health Care: A Mixed-Methods Study

**DOI:** 10.1177/10748407211000050

**Published:** 2021-04-07

**Authors:** Susanna Pusa, Ulf Isaksson, Karin Sundin

**Affiliations:** 1 Umeå University, Sweden

**Keywords:** Family Systems Nursing, Family Systems Nursing conversations, family nursing, implementation, mixed methods, home health care

## Abstract

To support the incorporation of Family Systems Nursing (FSN) in clinical practice, more understanding is needed about the implementation of FSN in home health practice settings. Thus, the aim of this study was to evaluate nurses’ perspectives about the implementation process of Family Systems Nursing Conversations (FSNCs) in home health care. A mixed-methods research design was used, integrating qualitative and quantitative data, and using triangulation as a methodological metaphor. The Quality Implementation Framework (QIF) was applied to guide the implementation process, and Proctor et al.’s taxonomy of implementation outcomes was used to evaluate the process. The findings demonstrated that FSN implementation was in progress. Overall, acceptability and appropriateness of FSNCs were evaluated as positive by home health nurses; however, some obstacles were found relating to feasibility, adoption, and fidelity. These results contribute to an increased understanding of the process and challenges of implementing FSNCs in home health care.

In the literature, there is a need to bridge the gap between available knowledge and knowledge from applied outcomes in health care (Campbell et al., 2007; McQueen, 2001). In particular, there is a need for more research on how to facilitate the incorporation of Family Systems Nursing (FSN) in clinical practice settings (Bell, 2016; Duhamel, 2017). The concept of implementation has been described as a process of putting into operation or integrating new practices within a specific setting (Greenhalgh et al., 2004; Nilsen, 2015). FSN is an approach in which nurses prioritize and support family health. A nurse who practices FSN chooses practices that keep the family system in mind and that offer possibilities for health and healing of families. This implies paying attention to the individual, the relationship between two or more family members, and/or the relationship between the family and the health care provider(s), the health care system, society, and/or culture. FSN is based on the perception that “illness is a family affair” (Wright & Bell, 2009, p. ix); consequently, all family members mutually influence each other. Thus, if one family member suffers from an illness, the other family members are also affected (Wright & Leahey, 2013). Supporting families through Family Systems Nursing Conversations (FSNCs)—nurse-led family conversations with an FSN orientation—can promote health and address illness suffering of the whole family (Dorell, Bäckström, et al., 2016; Östlund et al., 2016; Sundin et al., 2016).

Studies in the field of FSN have shown positive outcomes for both families (Östlund & Persson, 2014; Sigurdardottir et al., 2017) and nurses (Dorell, Östlund, & Sundin, 2016; Svavarsdottir et al., 2018; Voltelen et al., 2016). For families, FSNC can result in positive changes in cognitive, affective, and behavioral family functions (Östlund & Persson, 2014). In addition, research has found that families preferred an opportunity to receive FSNC support from nurses early in the illness process when the patient was still living at home (Dorell, Bäckström, et al., 2016). For nurses, education in FSN and FSNCs can lead to valuing families’ importance and involvement in nursing care (Broekema et al., 2018), as well as function as a professional competency to support families (Dorell, Östlund, & Sundin, 2016).

Implementation projects in clinical settings, which have used an FSN approach, have been identified in other countries and in a variety of health care contexts. For example, FSNCs have been implemented throughout the entire hospital system in Iceland in the Landspitali University Hospital Implementation Project (Svavarsdottir, 2008; Svavarsdottir et al., 2015). FSNC implementation projects have also been conducted in Denmark in heart failure units (Voltelen et al., 2016); in Canada, in psychiatric settings (Leahey & Harper-Jaques, 2010) and in pediatric settings (Martinez et al., 2007); in Sweden, in pediatric oncology settings (Marklund et al., 2018); in the Netherlands in a variety of health care contexts (Broekema et al., 2018); in Switzerland, in obstetric, gynecological, and neonatology care (Naef, Kaeppeli et al., 2020; Naef, Klausler-Troxler et al., 2020); in Germany, in oncology care (Zimansky et al., 2020); and in Hong Kong, in psychiatric care (Simpson et al., 2006; Wong, 2014). These studies have mainly focused on increasing nurses’ skill development in FSNC and how this new knowledge and skills influenced nurses’ perceptions and attitudes toward families.

When implementing an intervention in a clinical setting, process evaluation is important to assess and increase understanding of the implementation process (Proctor et al., 2011), and also to learn how and why clinical outcomes are reached or not (May et al., 2007). Proctor et al. (2011) argued that if an intervention is not well implemented, it will not be effective. Thus, process evaluation and confirmation of implementation outcomes must be performed prior to drawing reliable conclusions about the effectiveness of an intervention. Duhamel (2017) suggested that evaluating, in this instance, FSN education on family outcomes is a conceptual leap, with the risk of causing an unclear link between education and clinical outcomes (family outcomes) to emerge. This is because the nurses’ practices that could be linked to family outcomes remain unclear if not evaluated. Thus, the practices connected to FSN must be evaluated and described before the family outcomes. Nilsen (2015) has described how evaluation frameworks can be used when evaluating implementation to specify aspects of the implementation connected to successful implementation.

Consequently, implementation is more than merely offering an educational intervention in FSN with subsequent evaluation of outcomes for nurses, patients, and families. The purpose of the implementation is to change the practical work in the clinic setting, which is a complex process (Duhamel, 2017). The researcher should, therefore, systematically account for numerous processes (Bell, 2014). Only a few studies were identified that had this broad implementation focus on factors, aspects, and circumstances that, according to the nurses, affected the possibility of clinically adopting an FSN approach (Braun & Foster, 2011; Duhamel, 2017; Duhamel et al., 2015; Naef, Kaeppeli et al., 2020; Naef, Klausler-Troxler et al., 2020; Voltelen et al., 2016). A recent literature search of the scientific databases did result in identifying one study with a focus on implementation of FSN in the context of home health care (Petursdottir et al., 2019). The study by Petursdottir et al. (2019) focused on impact of an educational intervention in FSN within a specialized palliative home care unit at a university hospital. No studies were found in the context of municipal home health care.

More knowledge is needed to increase understanding about the implementation of FSNCs in home health care contexts from nurses’ perspectives. Thus, the aim of the study was to evaluate the implementation process of FSNCs in home health care from the nurses’ perspective.

## Method

In this study, a mixed-methods research design was implemented, using both an inductive and deductive approach. Qualitative and quantitative data were integrated and triangulation was used as a methodological metaphor (Erzberger & Kelle, 2003; Östlund et al., 2011).

### The Implementation Process

Process models can be used to guide the implementation process (Nilsen, 2015). In the present study, the Quality Implementation Framework (QIF; Meyers et al., 2012) was applied to guide the implementation process. The QIF outline is comprised of four phases: (a) initial considerations regarding the host setting, (b) creating a structure for implementation, (c) ongoing structure once implementation begins, and (d) improving future applications. The four phases consist of strategies that are aimed at supporting implementation. In total, there are 14 critical steps that provide practical guidance. When implementing interventions in clinical practice, effective education is described as one of the critical steps (Meyers et al., 2012). The research group for the present study designed a web-based education in FSN and FSNCs for nurses. The FSNC intervention and also the content and the structure of the web-based education are described in Table 1 and furthermore described in Pusa et al. (2019).

**Table 1.** Overview of the Basis in FSNCs and the Web-Based Education in FSNCs.

*The Intervention—FSNCs*
FSNCs are based on the CFAM, the Calgary Family Intervention Model (Wright & Leahey, 2013), and the Illness Belief Model (Wright & Bell, 2009). During FSNCs, the nurse takes a systemic approach, focusing on the interactions and relationships between family members. Narration and reflection are seen as essential tools in strengthening health and promoting healing by making beliefs of the situation visible (Benzein et al., 2008; Östlund et al., 2015).
*Web-Based Education*
The content in the education was based on previous research on FSNCs, with FSN as a foundation (Bell & Wright, 2015; Benzein et al., 2008; Östlund et al., 2015; Wright & Leahey, 2013). Central elements in the education course include systems theory (Bateson, 2000), communication theory (Watzlawick et al., 1974), and reflection theory (Ricoeur, 1992), all of which are key features of FSN (Wright & Leahey, 2013). The web-based education consisted of online web-based learning and two face-to-face seminars led by two persons from the research group. The web-based education included online learning material such as video lectures and written documents, plus two face-to-face seminars led by two persons from the research group. The purpose was to create a variety of learning elements, thus allowing multiple choices of knowledge acquisition. The first seminar was included in the beginning of the educational period and the other seminar in the end. Activities and content in the seminars consisted of introduction, discussions, and practicing FSNCs in the form of role-play. The content and the structure of the course are described further in more detail in a previously published study (Pusa et al., 2019).
*Practical Application*
Previous FSNC interventions have described a variation in the structure regarding number of conversations, length of the conversations, number of nurses in each conversation (i.e., one or two), included family members, and whether or not a closing letter was sent out to the families afterward. In this study, FSNCs in home health care were intended to be conducted by a single nurse with approximately two to three conversations with each family. No closing letters were included, and the reason and motivation for this were due to the fact that the relation and cooperation between the nurse and the families continue even after the series of structural FSNCs. The length of each conversation is estimated to take approximately 30 min. With an eye to the reality that each family and situation is unique, the intervention is kept flexible and allowed for the possibility of varying numbers of conversations and duration. During the FSNCs, each family member is invited to share their story about how he or she experience the family’s situation. The focus is to progress toward reducing the family’s suffering by strengthening beliefs considered to be facilitative and modifying beliefs considered to be constraining. Core components of FSNCs are as follows (Östlund et al., 2015): • Jointly reflecting with the family on expectations of the conversation series • Exploring the family structure • Ensuring that all family members are given space within the conversation and the opportunity to narrate their experiences • Collectively prioritizing which problem(s) most need to be discussed • Exploring significant parts of family narratives • Using reflective questions • Using appropriately unusual questions and challenging family beliefs • Giving commendations and acknowledging suffering • Inviting family members to reflect on each other’s narratives • Offering the nurse’s reflections

*Note.* FSNCs = Family Systems Nursing Conversations; CFAM = Calgary Family Assessment Model; FSN = Family Systems Nursing.

To evaluate implementation outcomes in this study, Proctor et al.’s (2011) taxonomy of implementation outcomes was used. For an overview of the application of Meyers et al.’s (2012) four phases of QIF and Proctor et al.’s (2011) implementation outcomes, see Table 2.

**Table 2.** Overview of the Application of Meyers et al.’s (2012) Four Phases of the QIF and Proctor et al.’s (2011) Taxonomy of Implementation Outcomes.

*Phase 1: Initial Considerations Regarding the Host Setting*
During the first phasein the QIF (Meyers et al., 2012), the home health care setting was assessed regarding needs, resources, fit, and capacity for implementing FSNCs, relying on knowledge gained by previous studies in FSN and on discussions of needs with persons in leading positions within the relevant administration. In addition, possibilities for adapting the FSNC intervention were assessed. Approval for the implementation of FSNCs was provided by the municipal management, administration, and the head of home health care. “Effective pre-innovation staff training” (Meyers et al., 2012, p. 470) is one critical step in the first phase. As such, the research group designed a web-based education in FSN and FSNCs for nurses in municipal home health care. Content and structure regarding the web-based education are described further in Table 1. During the implementation process, adjustments to the structure of FSNCs were made in accordance with clinical conditions and the nurses’ suggestions.
*Phase 2: Creating a Structure for Implementation*
An implementation team was created and an implementation plan was developed. The implementation team consisted of the research group collaborating with managers for home health care. The research group consisted of researchers with documented knowledge and previous experience in both FSN and applied research methods, one (K.S.) of whom was a qualified family nursing educator with extensive experience in researching and teaching FSN.
*Phase 3: Ongoing Structure Once Implementation Begins*
The nurses were offered support through the implementation process, both during the education and during the implementation. The nurses were also offered a professional that supported them theoretically and practically when needed. Regarding process evaluation, Proctor et al.’s (2011) taxonomy of implementation outcomes was used. In the present study, five of Proctor’s eight implementation outcomes were utilized: 1. Acceptability, if and how the clinical intervention being implemented is agreeable, palatable, or satisfactory. 2. Appropriateness, the perceived fit, relevance, or compatibility of the clinical intervention being implemented. 3. Feasibility, the extent to which the clinical intervention being implemented can be successfully used or carried out. 4. Adoption, the intention or action to try or employ the clinical intervention, also referred to as uptake. 5. Fidelity,the degree to which the clinical intervention is being implemented as intended.
*Phase 4: Improving Future Applications*
By doing this study and by using Proctor et al.’s (2011) taxonomy of implementation, the findings contribute to learning from experience, the *fourth phase*in the QIF (Meyers et al., 2012).

*Note.* QIF = Quality Implementation Framework; FSNCs = Family Systems Nursing Conversations; FSN = Family Systems Nursing.

### Theoretical Proposition

The theoretical proposition concerning the implementation of FSNCs in home health care was that it would be possible to implement FSNCs in the clinical setting. Through education, collaboration, and by following a structured implementation plan, nurses’ attitudes and approaches toward the inclusion and support of families would be enhanced. The success of the implementation would be influenced by personal, social, practical, and organizational aspects.

### Participants and Setting

The participants consisted of municipal primary health care nurses and municipal registered nurses employed in municipal home health care in a middle-sized municipality in northern Sweden. In Sweden, home health care includes both nursing care in specific residential homes and nursing care for people living in their own homes. In this study, the focus was on home health care provided by nurses to people living in their own homes and requiring care service. The nurses were assigned to different geographical areas, where they were responsible for providing multidimensional care for persons enrolled in the home health care program. There was no age limit for receiving home health care; however, most people enrolled were persons 65 years or older. Twenty-seven nurses completed the web-based education in FSN and FSNCs on two different occasions (referred as Groups 1 and 2), including 16 who answered the pre- and post-questionnairesand 14 who were interviewed. Of the 27 nurses, 16 nurses were still employed in the home health care at the time for data collection, and 14 nurses had changed workplace or retired. For an overview of participants’ characteristics, see Table 3.

**Table 3.** Overview of Participants’ Demographics.

Gender	
Female	15
Male	1
Age (median [minimum–maximum])	40 (24–56)
Number of years working as a nurse in home care	
≤3 years	13
4–9 years	1
≥10 years	2
Number of years working as a nurse	
≤3 years	2
4–9 years	4
≥10 years	10
Number who have education at master’s level	10

### Ethical Considerations

The study was approved by the Research Ethical Board (2014-235-31Ö) and was conducted in accordance with the Helsinki Declaration for Human Research (World Medical Association, 2013). Both written and verbal information regarding study aim, voluntary participation, and confidentiality were provided to the participants. All participants gave their written consent to participate in the study.

### Data Collection

#### Qualitative data collection

**Figure 1.**
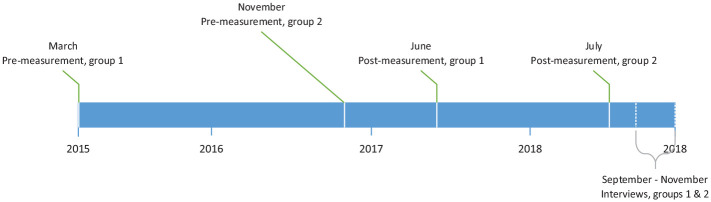
Time frame for data collection.

Individual, semistructured interviews were conducted between September and November 2018. A time frame for the data collection is presented in Figure 1. All interviews were conducted by the first author, and the nurses selected a time and location that was suitable for them. The interviews lasted between 18 and 40 min, with an average of 32 min. An interview guide, with open-ended questions, was used to encapsulate the nurses’ perceptions and experiences of the implementation. The questions focused on the nurses’ perceptions of if and how they used the FSN approach and FSNCs. The questions then further explored the nurses’ perceptions of what made FSNCs more or less suitable, their experiences of conducting FSNCs, aspects that influenced or affected the implementation, experiences of support during the implementation process, and perceptions of the FSNC content. Follow-up questions were used to clarify and to gain more information.

#### Quantitative data collection

Nurses’ attitudes about the importance of families in nursing care were measured with the Families’ Importance in Nursing Care–Nurses Attitudes (FINC-NA) questionnaire (Saveman et al., 2011). Data were collected both prior to the web-based education and 20 to 27 months after completion of the web-based education (see Figure 1). The instrument consists of 26 items, which are scored on a 5-point Likert-type scale and range from 1 (*totally disagree*) to 5 (*totally agree*). FINC-NA has four subscales: (a) family as a resource in nursing care (FAM-RNC), (b) family as a conversational partner (FAM-CP), (c) family as a burden (FAM-B), and (d) family as its own resource (FAM-OR). Scores for the FAM-B subscale were reversed for analysis, as the items were negative statements about the family. Higher scores indicated more positive attitudes. In addition, the instrument included one generic question with a 4-point Likert-type scale with a range from 1 (*very negative*) to 4 (*very positive*) concerning the nurses’ overall attitudes toward families’ involvement in nursing care. Additional questions concerning participants’ demographic information were also collected, which included age, gender, years of employment, and if severe illness in one’s own family had been experienced by the participant.

### Data Analysis

#### Qualitative data analysis

The audio-recorded interviews were transcribed and analyzed using a deductive qualitative content analysis that was performed in several steps (Elo & Kyngäs, 2008). First, a structured matrix for the analysis was developed, based on the five Proctor et al. (2011) implementation outcomes that were used in the study: acceptability, appropriateness, feasibility, adoption, and fidelity (see Table 2). In the next step, the text was read through repeatedly, and all interview data were reviewed for content. Thereafter, meaning units were identified in accordance with the aim of the study and were sorted into the predetermined categories (i.e., implementation outcomes). Subsequently, meaning units within the categories were condensed and labeled with codes. In the following step, coded meaning units were grouped into subcategories based on differences and similarities (Elo & Kyngäs, 2008). Two of the predetermined categories, adoption and fidelity, were combined in one category, as the content of the two overlapped with striking similarity.

#### Quantitative data analysis

Descriptive statistics are presented in mean scores, standard deviations, medians, and interquartile ranges (IQRs), when applicable. Comparisons between pre- and post-data were analyzed using Wilcoxon’s signed-rank test and using Rosenthal’s (1994) formula to calculate the effect size. According to Wolf (1986), effect sizes over 0.25 are considered as educationally significant, or that something was learned, and effect sizes over 0.50 are considered as practically/clinically significant, or that something changed. A *p* value of <.05 was considered statistically significant. One hypothesis of the study was that education in FSNCs would have a positive effect on nurses’ attitudes toward families’ importance; subsequently, a one-tailed *t* test was used to analyze the whole group. However, a two-tailed *t* test was used to analyze subgroups, as we were not clear about which group had improved. Age was divided into two groups based on a median of 40 years. Data analyses were performed using SPSS, Version 25, and Excel 2016.

#### Integration

Triangulation as a methodological metaphor was used when the qualitative and quantitative findings were integrated. The method is described by Erzberger and Kelle (2003) and further exemplified by Östlund et al. (2011). The triangulation links the theoretical proposition to the empirical findings, and different data sources are viewed as equally important. The sides of the triangle represent the relationships between the theoretical proposition and the empirical findings from qualitative and quantitative data. The integrated findings can be interpreted as convergent, complementary, and/or divergent.

## Findings

The findings are presented in three sections: the findings from the interviews with the nurses, the results from the FINC-NA questionnaire, and the integration of the empirical findings with the theoretical proposition.

### Qualitative Findings

The qualitative findings are presented in four categories, by means of the matrix used during the deductive analysis: acceptability, appropriateness, feasibility, adoption and fidelity. The acceptability category concerned the nurses’ perceptions and experiences regarding FSNCs in relation to usability (e.g., performance experience/ease of use) and perceived value, including aspects influencing acceptance. Appropriateness was about the nurses’ perceived suitability, relevance, and compatibility of FSNC in the context (i.e., to nurses, families, and overall in the setting home health care). Feasibility was about perceptions and influences on the practicability of FSNCs (i.e., aspects influencing the success of the implementation connected to feasibility). Adoption and fidelity described if and how the nurses were working with FSN, with connection to fidelity. For an overview of categories and subcategories, see Table 4.

**Table 4.** Overview of Categories and Subcategories.

Categories	Subcategories
Acceptability	• Fluctuating usability • A supportive approach • Reciprocal influence within the work team
Appropriateness	• Suitable for the nursing profession • Suitable for home health care • Suitable for families
Feasibility	• Competence comes with practice • Need for adjustable technique and local conversations • Heavy workload as a hindrance • Essential with supporting managers and working teams
Adoption and fidelity	• Using family conversations as intended • Using family conversation in a modified way

#### Acceptability

##### Fluctuating usability

The perceptions of ease of use varied among the nurses and over time. Experiences and expectations emerged where FSNCs were perceived as easy to use with their clear goals and descriptions. The nurses reported that the conversations facilitated communication with families, which improved the co-creation of plans and goals. Furthermore, working and communicating with families in this way was perceived as time saving, as problems within the family could be solved, and strategies to handle the situation and the future could be envisioned. As one nurse stated,

Well, it’s good, like, it makes it easier. Hopefully, they find a solution themselves and it’ll be less work for me that way if they can, together in the family, solve a problem or find good strategies.

Some nurses described using FSNCs as a different way of working than how they were used to working and communicating, which was challenging. Adopting more of a listening role was not easy, as they were mainly used to giving advice. Other nurses experienced the conversations as an easy and natural way of working toward the role as conversation leader. However, if they were insecure, they perceived the conversations and their role as a conversation leader as more complex. The usability of FSNCs improved over time, especially when the content and form of the FSNCs were changed and adapted to clinical setting (i.e., no closing letter and no fixed number of conversations). The nurses described how their acceptance and experience of effort improved when conducting FSNCs, as they found it to be less complicated and demanding than they initially expected.

##### A supportive approach

The nurses described how FSNCs could be beneficial for creating a supportive relationship with increased openness between the nurse and the family. After conducting FSNCs, they described how they more clearly saw the benefits of this type of family conversation. This new way of working and thinking was considered by the nurses to have several possible benefits and value for both the nurse and the family. The FSNCs were perceived as having the power to encourage families to share their stories with each other and to promote support within the family. Furthermore, FSNCs were seen to increase understanding within the family and awareness about possible strengths and resources within, and outside, the family. One nurse offered,

I think it’s a great idea, all of it; you might wish that everyone gets the opportunity to, like, take part in this, that one could include even more how to talk and solve situations.

##### Reciprocal influence within the work team

The acceptance of FSNCs varied among the nurses and over time. Individual perceptions were also affected and influenced by coworkers’ perceptions. This social influence could affect the acceptance of FSNCs in multiple directions, having the power to reduce or increase perceived benefits. Limited influence and codetermination in the initial phase of the implementation, before beginning web-based education in FSNCs, and being required to complete the web-based education were seen as adversely affecting attitudes and acceptance. A desire was expressed for increased involvement and participation in decisions before implementing FSNCs, which was seen as important to plan and mentally prepare for the implementation. As one nurse suggested,

To start with, there were many negative thoughts about this, of course you get affected. Over time, you sit during the coffee breaks and talk about this, and then you get another view from colleagues. Then you think, oh my God, maybe it’s good after all.

#### Appropriateness

##### Suitable for the nursing profession

The nurses perceived the FSNCs to be compatible with their profession as a nurse in several ways. FSNCs were perceived as compatible with the nurses’ desire to support health, and learning and leading FSNCs were suitable because the nurses already had experience in communicating. FSNCs were also seen as compatible with the nurses’ goal to provide good nursing care and support, because FSNCs were a tool to create shared visions and plans with the family regarding their care and how to view the future. As one nurse stated,

It’s a strength when you’re gonna try to have these conversations, as a nurse it’s absolutely a good method, I think.

##### Suitable for home health care

Implementing FSNCs were perceived as appropriate and suitable for home health care based on various aspects. FSNCs were experienced as a way to pay attention to family members’ experiences and needs, which otherwise were at risk of being overlooked. FSNCs were seen as more suitable when the patients were enrolled in home health care for the long term, compared with when the patient received home health care for a short term and/or when home health care was only responsible for some aspects of the care (i.e., shared nursing responsibility with health care centers). One nurse suggested that FSNCs are

. . . especially [suitable] if they have a close relative who just got enrolled in the home health care, the fact that this person has a need for more help it can really be tough for everyone close to the ill person. It is suitable as one gets to meet relatives and listen to their experiences.

##### Suitable for families

FSNCs were seen to be suitable for most families, although some nurses found the conversations to be more valuable or useful for families with visible communication problems or disagreements. The nurses experienced a need and desire from family members to be acknowledged and to be more included in the care of their relative. As one nurse noted,

Well, I think that the concept is good, absolutely. I do believe there are many who need this moment; many relatives can have a need for it. I really think so, so the concept I think is good.

#### Feasibility

##### Competence comes with practice

The findings show some variations in the nurses’ perceived competence and knowledge in terms of leading the FSNCs. The web-based education prior to the implementation was seen as a prerequisite to gain sufficient knowledge to support families through FSNCs. In addition, having access to the education material and the guide for the conversations was perceived as supporting and contributing to their feelings of self-confidence. Still, the nurses’ confidence and trust in their ability to lead FSNCs varied. A range was found, between trusting their ability, in which supporting families felt natural, and fearing being unable to handle distressed feelings and situations. Some kind of FSN continuous development was suggested to update knowledge and skills. Furthermore, the nurses shared that they felt pressure to deliver something “useful” for the families during the conversations. The nurses experienced that feeling secure and confident in performing FSNCs increased as they practiced carrying out the conversations, and their nervousness and performance anxiety reduced. Discussing FSNC content and execution with colleagues was seen as educational, contributing to new perspectives and leading to an increased understanding of FSNCs by learning from each other. As one nurse stated,

Well like, the first time, it felt like, well, it’s kind of awkward, and it feels like you’re really thinking now I’m gonna say this and then I’m gonna do that, so it becomes stilted somehow. But then when you’ve done one [conversation] it feels like it becomes more natural.

##### Need for adjustable technique and local conversations

The nurses reported that they needed more access to technical aids in the form of speakerphones to be able to include family members who lived far away. It was considered natural to primarily have the FSNCs in the patient’s home; however, on some occasions, the nurses needed to conduct the FSNCs at a facility outside the patient’s home. This was requested when the patient had difficulties participating in the conversation, such as severe physical or cognitive impairment. As one nurse suggested,

Many times the patient could be so, well, extremely demented or in such bad shape that they are unable to participate. Either they might be too tired to keep up with the conversation, or they’re so cognitively impaired, so they don’t know what it’s about. And then you might wish that maybe there was some other place to sit, a room for conversations.

##### Heavy workload as a hindrance

A stressful work environment with a heavy workload was described as hampering the practice with families. However, this varied over time, with periods of opportunity for offering and conducting family conversations. A resource nurse was available for the nurses when they needed to have someone cover for them, a practice that could facilitate the feasibility of FSNCs. However, this resource was seen as hard to use, as the resource nurse was often booked to do other nursing tasks and plans had to be rescheduled whenever one colleague was absent from work. At the beginning of the implementation, the nurses described some instability in the organization in terms of staff, employment, and lack of routines, as municipal home health care was a fairly new organization. One nurse suggested,

Some periods have been really hard; then it simply hasn’t been on the to-do list, when you barely have time to eat lunch or go to the bathroom, it goes without saying. But when there’s been calmer periods, then there’s been all the possibilities in the world to include these conversations in the nursing care.

##### Supporting managers and working team essential

Experiencing support and having committed managers were described as essential for the implementation and feasibility of FSNCs, as this motivated the nurses to prioritize FSNCs. The perceived amount of support from management varied, from perceptions of insufficient support and commitment to satisfaction with management support. Establishing commitment throughout the whole working team, across all managers and colleagues, was perceived as important and sometimes crucial for a successful FSNC implementation. To increase feasibility, the nurses needed forums with colleagues and with their closest manager to receive support through discussing, reflecting, and planning together. Furthermore, the nurses wished for allocated time and more resources, at least in the initial stages, until they felt more experienced and skilled in leading the conversations. In addition, the support from the implementation team was considered sufficient, and furthermore, that adjustments had been carried out in accordance with their suggestions, which the nurses reported had positively affected the feasibility. As one nurse stated,

Yes, well I don’t really know how that would work but, together with your manager, you try to figure out how are we are gonna work with this. How are we gonna make it happen for it to be ongoing. Like be with us all the time and not just now when we’re doing it but we’d continue and see something in the long term in all this.

#### Adoption and fidelity

##### Using family conversations as intended

The nurses described using theory-based core components of FSNCs, which they learned in the web-based education. The nurses described exploring the family structure by asking the family’s perception of the family to obtain an overall picture of the family structure and the different roles within the family. Ensuring all family members are given space within the conversations and have the opportunity to narrate their experiences was highlighted as important, as it enabled the family to gain an understanding of each other’s experiences. Furthermore, the nurses engaged in collaboratively prioritizing which problem/problems most needed to be discussed, where they used reflective questions and invited family members to reflect on each other’s narratives to stimulate the family member’s awareness of their beliefs. The nurses also described giving commendations and acknowledging suffering within the families. Of a total of 14 nurses, seven described conducting family nursing conversations in the intended way; in other words, they had approximately two to three conversations and followed the consecutive content order for the different conversations. An intention to continue “thinking family” was experienced. Some nurses had offered FSNCs to families, but the families declined the offer. Regarding continuing to use FSNCs in their practice, one nurse offered,

I will absolutely continue, at least the way of thinking and all that, but also try to manage having two or three conversations because there’s definitely a need for this for many people.

##### Using family conversations in a modified way

The nurses shared that they used the knowledge from the web-based education in other ways than the prescribed structure of having a conversation series with the whole family. Following the education, the nurses described considering and applying a more family-centered approach to their practice. The nurses embraced a different way of thinking and acting where they adopted the power of taking a step back, remaining silent, and listening when interacting and communicating with families. The nurses described trying to encourage family members to listen to each other to discover and understand the different opinions and experiences of the situation within the family.

Even if the nurses saw the family as important before the implementation, they described applying a new approach to interacting with families, with a new perspective and new ways to communicate, listen, and ask questions. The nurses described using elements from the web-based education when meeting some or all of the family in different contexts, such as using open-ended questions with a family member when having pharmaceutical conversations, health care planning meetings, and during the first meeting when the patient was enrolling in home health care. As one nurse noted,

I think it’s been great to have this knowledge; it’s with you all the time. I’ve had good use of it because I think in a whole different way than before actually, making relatives a part of my work as a primary health care nurse.

Some nurses received new insight and considered the family as a unit. The nurses described better understanding about the uniqueness and influences within every family, with increased insight about the value of trying, when possible, to involve the whole family and not only the patient and his or her closest relative. They described the importance of gaining knowledge about how the family members viewed themselves and the family structure. The nurses shared that they communicated differently with family members after the education, trying to involve as many family members as possible. The nurses described how they adopted a way of working with families to obtain their own resources and the ability to develop their own solutions to hardships, with support from the nurses. Furthermore, the nurses described trying to support the family members by inviting self-reflection about what was best for them, instead of giving answers to the families.

### Quantitative Findings

The findings demonstrated an increase in all subscales, including total score; however, the pre/post difference was not significant (see Table 5). In two subscales, FAM-CP and FAM-OR, effect sizes showed educationally significant differences between baseline and follow-up. Furthermore, the results indicated that the nurses’ overall attitude to family involvement in nursing care changed over time, *Mdn*. 3 (IQR = 3–3) versus 3 (IQR = 3–4), *p* = .227, effect size = 0.20. However, this increase was not statistically significant.

**Table 5.** Differences Over Time in Nurses’ Attitudes Regarding Families’ Importance in Nursing Care.

Variables	Distribution measures	Baseline	Follow-up	*p* ^a^	Effect size
FAM-RNC	*M* (*SD*) *Mdn* (IQR)	40.19 (4.48) 41.5 (37.0–44.5)	40.69 (6.34) 42.0 (35.5–46.0)	.294	0.211
FAM-CP	*M* (*SD*) *Mdn* (IQR)	28.00 (5.11) 27.5 (23.3–33.8)	30.19 (4.96) 30.5 (26.0–34.5)	.062	0.277
FAM-B	*M* (*SD*) *Mdn* (IQR)	14.81 (3.06) 16 (12.5–17.0)	15.69 (3.55) 16.0 (13.3–18.8)	.230	0.137
FAM-OR	*M* (*SD*) *Mdn* (IQR)	13.88 (2.70) 13.5 (12.0–16.0)	15.12 (3.22) 15.0 (13.0–18.0)	.078	0.256
FINC-NA TOT	*M* (*SD*) *Mdn* (IQR)	96.88 (11.00) 95.5 (88.3–107.5)	101.69 (14.12) 101.0 (88.8–115.5)	.294	0.041

IQR = interquartile range; FAM-RNC = family as a resource in nursing care; FAM-CP = family as a conversational partner; FAM-B = family as a burden; FAM-OR = family as its own resource; FINC-NA = Families’ Importance in Nursing Care–Nurses Attitudes; TOT= total score.

a One-tailed *t* test.

A subgroup analysis was performed to establish which group had been influenced most by the implementation. This was done using a two-sided significance test. The results showed that nurses who had no personal experience of severe illness in their family increased in two subscales, FAM-CP, *M* = 25.71 (±4.15) versus *M* = 31.57 (±4.96), *p* = .028, FAM-RNC, *M* = 37.57 (±3.10) versus *M* = 42.00 (±5.03), *p* = .027, and on total score, *M* = 90.57 (±8.10) versus *M* = 103.86 (±14.65), *p* = .018, between pre- and post-test. FAM-CP includes considering the family as a conversational partner, finding out who belongs to the patient’s family, and discussing with the family when planning nursing care. FAM-RNC means considering families as a resource in nursing care, valuing the presence of the family in nursing care.

When comparing between age groups, the results showed that over time (i.e., pre- and post-comparisons), nurses 40 years or younger saw families as a burden, FAM-B, *M* = 15.44 (±2.96) versus *M* = 13.89 (±3.02), *p* = .026, at a higher rate compared with nurses 41 years or older, who saw families less as a burden over time, *M* = 14.00 (±3.21) versus *M* = 17.00 (±2.89), *p* = .018.

### Integration of the Findings

**Figure 2.**
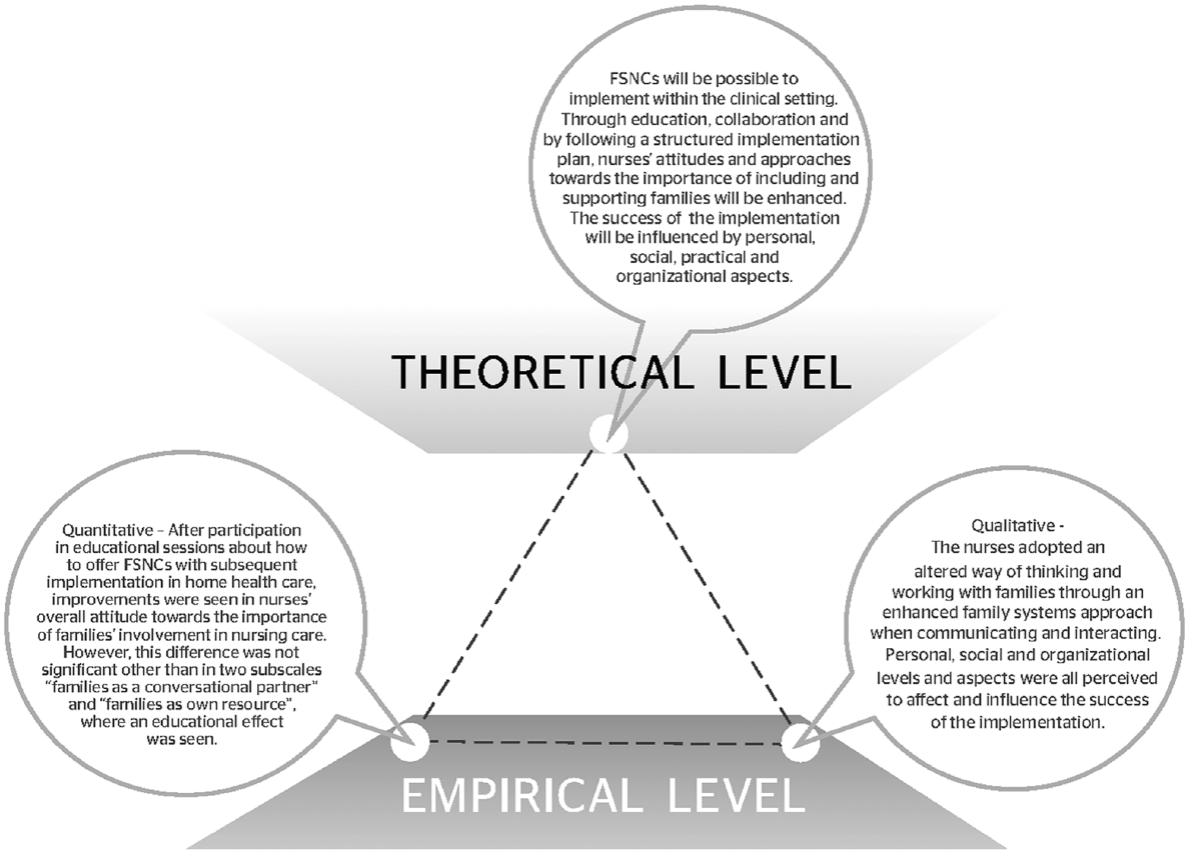
Triangulation diagram of the logical relationship between the theoretical proposition, the qualitative findings from the interviews, and the quantitative data from the FINC-NA questionnaire. *Note.* FINC-NA = Families’ Importance in Nursing Care–Nurses Attitudes.

The integration of qualitative and quantitative empirical findings, together with the suggested theoretical proposition, is illustrated as a triangle metaphor. The integration is then further elaborated in the text. In this study, the quantitative results and the qualitative findings were interpreted as being mostly convergent but partly complementary. The empirical results were in line with the theoretical proposition (see Figure 2).

The theoretical proposition suggests that it is possible for nurses to learn FSNCs and to implement an FSN approach in home health care. This is supported both by the quantitative and the qualitative findings. The quantitative results found that nurses’ overall attitudes toward the importance of family involvement in nursing care increased over time. In two subscales (FAM-CP and FAM-OR), the change over time showed an educational improvement, indicating that something had been learned. Nurses were more likely to consider the family as a conversational partner and to see the family as its own resource. This implies that the nurses were able to involve the family in discussions and in planning nursing care. Furthermore, nurses now viewed families more as a resource and utilized the families’ strengths and resources in their care of the family.

The qualitative results also support this part of the theoretical proposition, as the nurses expressed applying a more FSN approach to their working, thinking, and interacting with families, and more readily acknowledging the family as a unique unit with its own resources. This resulted in an increased willingness to include families in their nursing care and invited a different way of communicating, with the nurse doing more listening and asking reflective questions. Even though the nurses did not always follow the intended structure of FSNCs, they used core components of FSNCs when meeting the family, or part of the family, in their daily practice.

The theoretical proposition further suggests that the findings may contribute to an understanding of what influences and affects FSNC implementation. This was supported only by qualitative data and thus considered as complementary findings. Personal, social, practical, and organizational aspects affected the success of the implementation to varying degrees. The perceived competence and confidence in conducting FSNCs varied, affecting the implementation in both directions. The implementation was positively influenced by the perception that FSNCs benefited and were suitable for the nursing profession, for families, and for home health care. Furthermore, having the opportunity and mandate to influence the adjustment of the FSNCs was perceived as important. Support from managers and from the working group was seen as essential for implementing FSNCs, while a heavy workload, combined with lack of time, was seen as a hindrance.

Complementary findings were also seen in the quantitative data regarding the subgroup results, as nurses who had no personal experience of severe illness within their own family reported significantly more positive attitudes regarding the importance of the family over time. In addition, over time, nurses 40 years or younger viewed families more as a burden, while nurses 41 years or older viewed families as less of a burden. These results were not found in the qualitative findings.

## Discussion

The findings of this study supported that FSNC implementation in home health care was in progress, even if not as fully implemented as intended. The nurses described using the FSN approach in their daily practice when meeting families or family members, but used structured FSNCs to a lesser extent. The integrated results showed that following the web-based education, nurses had increased FSN thinking and working. Nurses’ attitudes toward family importance and involvement in nursing care were initially positive and increased after the web-based education and along with the implementation, even though the change was not statistically significant. The most prominent change over time was seen in nurses’ attitudes regarding the family as a conversational partner and viewing the family as its own resource, which included supporting families in discovering and using their own strengths and resources. Overall, the results indicated a higher awareness and willingness to include families and working with the family system.

Fixsen et al. (2005) described that fidelity to the intervention/method implemented is one crucial aspect when evaluating implementation outcomes because fidelity, in this study, indicates whether the FSNCs were carried out as intended. This is important, as the intervention may have a limited impact if not properly implemented, although it has been argued that a positive impact can be accomplished even when the intervention is not implemented entirely as intended (Moore et al., 2015).

The findings in our study show that even if the nurses did not always follow the structure of FSNCs, they described using its core components when communicating with both patients and families. However, the structure of FSNCs (i.e., number of conversations, length of conversations, whether the whole family attends, etc.) is not viewed as a crucial factor for fidelity, as this structure of nurse-led conversations with an FSN approach differs across several studies. Previous studies of FSNCs describe variation in structure, from the 15-min or less family interview (Wright & Leahey, 1999) to a series of repeated conversations of varying length (Östlund & Persson, 2014). Furthermore, flexibility and adaptation of the structure could be achieved by adhering to a specific setting and conditions, as long as the systemic approach toward families was maintained. Thus, in terms of fidelity, it seems more important that the nurses in our study described developing knowledge and competence in central elements of FSNCs, such as viewing the family as a unit, working with an inclusive approach toward families, and encouraging open communication that promoted reflection, rather than rigidly following the structure in terms of number of conversations, length of conversations, and whether the whole family attends or just one or few family member/s.

The nurses in our study described an improved way of communicating with families, in which they embraced the power of taking a step back and adopting the power of silence. This is concordant with other studies (Dorell, Östlund, & Sundin, 2016; Gusdal et al., 2018), where nurses’ previous discomfort with silence subsided when practicing FSNCs. The impact of silence was seen as offering time for reflection.

The findings of our study underscore that FSNC implementation is influenced by multiple aspects to varying degrees, ranging from the nurses’ attitudes to practical, organizational, and social aspects. This is congruent with Nilsen (2015), who suggested that implementation entails a systems approach, because implementation outcomes are influenced within and across different levels and types of determinants. Thus, implementation is a multidimensional phenomenon, and determinants (factors influencing the implementation outcomes) occur at multiple levels, from the health care professional to the organization and beyond. Nilsen (2015) further indicated that four domains are usually represented in determinant frameworks that affect implementation outcomes: characteristics of the intervention, characteristics of the adopters, organizational context, and implementation process factors.

The characteristics of interventions or methods, including perceived advantage, complexity, compatibility within the setting, and the possibility of adapting the intervention to the clinical context and setting, affect implementation outcomes (Rogers, 2003). In our study, the nurses described the overall fit of FSNCs as compatible with their profession and useful for families. The adjustments made in the structure of FSNCs during the implementation, in collaboration with the nurses, were perceived to improve the suitability of the intervention. This influenced the acceptance and facilitated the implementation as perceived ease of use increased.

The findings in our study showed that, when practicing FSNCs, the nurses’ perceived competence grew with increased acceptance and willingness to continue working with families in this new way. In addition, when the nurses communicated with families in this manner, they saw the benefits for both themselves and the families. Previous studies have shown that working with an FSN approach can be professionally rewarding (Dorell, Östlund, & Sundin, 2016; Duhamel et al., 2015) and positively affecting nurses’ empathic abilities (Gusdal et al., 2018). Duhamel et al. (2015) described how nurses’ belief in the importance of FSN was related to the nurses’ beliefs about their own skills; when nurses learned more about how to support families, the perceived usefulness of FSN increased. This was also supported in our findings.

However, adopting the FSN approach was not always seen as easy, as for some, it was a different way of working and thinking compared with what they were used to. Fixsen et al. (2005) offer that the behavioral changes required for successful implementation are often challenging for most people to accomplish. From a feasibility perspective, the nurses in our study described the education prior to the implementation as a prerequisite for gaining sufficient knowledge; however, disparities were seen in the nurses’ confidence in their own competence to perform FSNCs. Some nurses, on the contrary, experienced the conversations and the FSN approach as natural, feeling secure in their role as conversation leader. However, feelings of insecurity, along with fears of not being able to handle distressed feelings, also were reported.

This is similar to the findings presented by Duhamel et al. (2015), where a fear of hurting feelings or being perceived by families as too insensitive was reported to affect nurses’ trust in their FSN skills, which could hinder FSN practice. Wright and Leahey (2013) described the fear of not being able to handle the situation and performance anxiety, when failing to solve problems, as beliefs that can hinder nurses when working with an FSN approach. For nurses to become skilled in the practice of FSNCs, hours of clinical practice are required, preferably with supervision and feedback (Bell, 2016). In addition, nurses need to obtain coaching and supervision to continue developing their skills (Bell, 2016), potentially in the form of some kind of mentorship.

Our study found that support from local managers, as well as from the implementation team and the working team, was essential to the feasibility and implementation of FSNCs, as it encouraged the nurses to prioritize the family conversations. This is similar to the findings of Duhamel et al. (2015), who suggested that institutional and managerial support is crucial for FSN integration. Other implementation studies have also described the importance of managerial support (Damschroder et al., 2009; Grol et al., 2007; Lukas et al., 2007). Similar to the findings in our study, Schmid (2010) argued that middle-level managers have the authority to influence implementation and should be seen as key players when it comes to implementation, as these leaders are able to either promote or hold back an implementation. Sandström et al. (2011) also asserted that it is particularly important that managers have the ability to create a context and culture in which research-based knowledge can be sustained. Leader characteristics that helped to promote implementation included the ability and willingness to provide feedback, to emphasize the importance of research-based work, and to lead by example.

The findings of our study highlight the importance of a context where a vision and commitment to FSNCs are shared by all, from managers to colleagues, and linked to feasibility. The nurses desired more opportunities to support each other and the implementation by discussing, reflecting, and planning together with colleagues and their closest manager. This is similar to Meyers et al. (2012), who described this social aspect as a process where formal and informal relationships can affect the success of the implementation and that consequently, this aspect should not be overlooked or neglected. Even Duhamel et al. (2015) described the importance of managers and nurses agreeing on the same philosophy to promote FSN implementation. Proctor et al. (2009) also suggested that implementation researchers must collaborate with community stakeholders because implementation research occurs in the real world in community-based settings.

### Clinical Relevance and Implications for Further Research

The implementation outcomes in this study are relevant to integrating FSN in clinical practice to improve the care for both patients and their families. Overall, our study contributes to the understanding of the complex process of implementing FSNCs in home health care settings. As support from managers was perceived as vital by the nurses to implement FSNCs, more research is needed that focuses on leadership and health managers’ beliefs and experiences related to FSNCs and the process of implementing FSNCs. To draw attention and to increase the understanding of FSN, educational material such as descriptions of core competencies for FSN (e.g., International Family Nursing Association, 2015; Östlund et al., 2016) could be distributed throughout health care settings to facilitate a context where FSN is valued and supported. Furthermore, future implementation of FSN in clinical settings should include multifaceted implementation strategies such as combining easily accessible customized education with recurring opportunities for reflections, practicing FSNC, and learning and supporting each other within the clinical setting.

From a research perspective, hybrid design studies would be appropriate for facilitating and speeding up FSNC implementation in a clinical setting. As hybrid design implementation studies combine elements of clinical effectiveness and implementation research, this can lead to more rapid and effective implementation strategies as well as valuable information for decision makers in health care (Curran et al., 2012).

### Methodological Considerations

A mixed-method design was used in this study, which is considered suitable when studying complex interventions and processes (Borglin, 2015). Combining qualitative and quantitative methods is a strength of our study, as we believe the study findings provide a more comprehensive picture of multiple aspects involved in the process of implementing FSNCs in a clinical setting.

The sample size of 14 nurses in the qualitative analysis was considered sufficient, as the interviews were rich in content and included a variety of responses. However, the sample size of 16 nurses in the quantitative analysis could be seen as a limitation, as the sample was rather small for statistical analysis. This limited the options when choosing statistical data analyses, and therefore, nonparametric test and effect size were eventually used due to concerns with the normality of the data. The FINC-NA instrument has been shown to be reliable and valid (Saveman et al., 2011), which adds strength to the study. Trustworthiness in the qualitative analysis was strengthened by continuous discussion of the coding scheme and the coding among the authors.

The taxonomy of Proctors et al.’s (2011) implementation outcomes is intended to be applied when studying and evaluating outcomes of implementation (Nilsen, 2015), and thus appropriate and suitable for the aim of this study. Three of the Proctors et al.’s (2011) eight implementation outcomes were excluded (penetration, sustainability, and costs). The reason for excluding costs was due to its inconsistency with the study purpose of looking at implementation outcomes from nurses’ perspectives. The implementation outcomes, penetration and sustainability, were excluded for the reason that these two outcomes should preferably be studied at a later stage in the implementation process.

## Conclusion

The findings of this study support that the FSNC implementation was in progress in home health care, even if it was not as fully implemented as intended. It is considered valuable that the nurses had somewhat altered their way of working and developed a new way of “thinking family” by being more inclusive and supportive to families. The process of implementing FSNCs was seen to be influenced by several aspects, which included support from, and involvement with, managers and coworkers; adjustments and adoption of the FSNCs model according to nurses’ preferences and the organizational setting; coaching and supervision to support continued knowledge and skills development; and creation of consensus within the organization with the common goal of working with an FSN approach. This study extends what is already known in the field of FSN implementation as it highlights implementation outcomes in the context of municipal home health care. This study contributes to the growing evidence about the processes needed to integrate FSN in clinical settings to ensure family nursing becomes “usual” care.
